# Correlation of Routine Admission Inflammatory Biomarkers with Individual Traumatic Brain Lesion Types in Mild Traumatic Brain Injury

**DOI:** 10.3390/biomedicines14020365

**Published:** 2026-02-05

**Authors:** Marios Lampros, Labrini Vlachodimitropoulou, Spyridon Voulgaris, George A. Alexiou

**Affiliations:** Department of Neurosurgery, University Hospital of Ioannina, 45 500 Ioannina, Greece; marioslampros@gmail.com (M.L.); labrinivlachodimitropoulou@gmail.com (L.V.); svoulgar@uoi.gr (S.V.)

**Keywords:** mild traumatic brain injury, biomarkers, inflammatory biomarkers

## Abstract

**Background**: Routine admission inflammatory and metabolic biomarkers have been proposed as adjunctive tools in mild traumatic brain injury (mTBI). However, their association with specific traumatic intracranial lesion types remains unclear. **Methods**: We conducted a prospective observational study including adult patients with isolated mTBI who underwent head computed tomography (CT) on admission. Admission laboratory parameters included the platelet-to-lymphocyte ratio (PLR), systemic immune-inflammation index (SII), and glucose-to-potassium ratio (GPR). Two predefined endpoints were assessed. The first compared biomarker values between CT-positive and CT-negative patients. The second evaluated associations between biomarkers and individual intracranial lesion subtypes, including analyses restricted to isolated lesions. **Results**: A total of 125 patients were included, of whom 95 (76%) were CT-positive. No significant differences were observed between CT-positive and CT-negative patients for PLR (*p* = 0.793), GPR (*p* = 0.531), or SII (*p* = 0.291). In lesion-specific analyses including all intracranial injuries, subdural hematoma (SDH) was associated with higher GPR compared with patients without SDH (*p* = 0.016). In analyses restricted to patients with isolated lesions, SDH was associated with higher PLR (*p* = 0.018) and higher GPR (*p* = 0.015). No significant associations were observed between any biomarker and intraparenchymal hemorrhage, subarachnoid hemorrhage, or epidural hematoma (all *p* > 0.05). Patients with multiple intracranial injuries exhibited higher PLR (*p* = 0.012) and higher SII (*p* = 0.021) compared with those with isolated lesions. After correction for multiple comparisons, none of the observed associations remained statistically significant. **Conclusions**: These findings suggest that routine systemic biomarkers have limited global discriminatory value in mTBI. Exploratory lesion-specific associations with SDH did not remain significant after correction for multiple comparisons, underscoring the preliminary nature of these findings.

## 1. Introduction

Mild traumatic brain injury (mTBI) affects millions of individuals worldwide each year and represents one of the most common reasons for emergency department (ED) visits, particularly among children and young adults [1]. Although most cases are managed conservatively, a clinically relevant subgroup of patients sustains traumatic intracranial injury and is therefore at increased risk of serious complications, including neurosurgical intervention, critical care admission, severe neurological deficits, or death. Computed tomography (CT) is the imaging modality of choice for detecting intracranial hemorrhage in mTBI [2]. Decisions regarding CT utilization are typically guided by validated clinical decision rules (CDRs), such as the Canadian CT Head Rule (CCHR), NICE guidelines, and the Pediatric Emergency Care Applied Research Network criteria (PECARN). While these tools demonstrate excellent sensitivity (approximately 95–100%), their specificity and positive predictive value remain low (5–10%), resulting in substantial overuse of CT imaging [2,3]. This overutilization contributes to ED crowding, increased healthcare costs, and unnecessary exposure to ionizing radiation, a concern that is particularly relevant in younger patients due to long-term malignancy risk [4].

To address these limitations, blood-based biomarkers have been introduced as adjuncts to existing CDRs [5,6,7,8]. Biomarkers such as glial fibrillary acidic protein (GFAP), S-100B, and ubiquitin carboxy-terminal hydrolase L1 have demonstrated the ability to maintain high sensitivity while reducing unnecessary CT scans by up to 30% [7,8,9]. Inflammatory mediators, including cytokines such as interleukin-6, have also been associated with symptom severity in mTBI [10,11,12,13]. However, the routine clinical implementation of these biomarkers is limited by high costs, reliance on specialized laboratory techniques, and reduced availability outside standard working hours. Consequently, increasing attention has been directed toward readily available, low-cost inflammatory biomarkers derived from routine blood tests, including the neutrophil-to-lymphocyte ratio (NLR) and platelet-to-lymphocyte ratio (PLR) [14,15,16,17,18].

On a recent study from our institution, we demonstrated that patients with CT-positive intracranial trauma or subdural hematoma had higher NLR and glucose levels compared with patients with other types of intracranial injury or concussion [15]. However, the potential roles of commonly studied routine inflammatory indices, including PLR, systemic immune-inflammation index (SII), and glucose-to-potassium ratio (GPR), remain insufficiently characterized, either for CT triage or for injury severity classification. Therefore, in the present study, we aimed to investigate the discriminatory performance and lesion-specific relevance of these biomarkers in patients with mTBI.

## 2. Materials and Methods

### 2.1. Patient Search and Study Design

We prospectively studied patients with isolated mTBI who were admitted to the neurosurgical department of the University Hospital of Ioannina over a one-year period from December 2024 to December 2025. Head CT imaging followed the Canadian CT Head Rule. Baseline blood samples were obtained at the time of admission at the emergency department. The University Hospital of Ioannina’s Institutional Review Board (IRB) approved the study (protocol code 28/22-11-2023 (θ.6)). This study was conducted and reported in accordance with the STROBE guidelines.

### 2.2. Inclusion Criteria

We included adult patients (≥18 years) who presented to the emergency department with isolated mild traumatic brain injury (mTBI) and Glasgow Coma Scale score 14–15, underwent head computed tomography, and had laboratory testing performed within 12 h of injury.

### 2.3. Exclusion Criteria

We excluded pediatric patients (<18 years), patients with diabetes mellitus (DM) or hematological disorders or malignancies because their laboratory values could be influenced by factors unrelated to mTBI. We also excluded patients suffering with extracranial injuries.

### 2.4. Data Collection

Patients’ demographic and clinical data were prospectively collected and entered into an electronic database. Recorded variables included age, sex, mechanism of injury, Glasgow Coma Scale score, type of intracranial traumatic lesion, and major comorbidities (diabetes mellitus, prior stroke, and coronary artery disease). The time interval between injury and blood sampling was estimated in minutes and documented. All laboratory analyses were performed in the hospital’s central clinical laboratory using standard routine assays.

### 2.5. Outcomes

The study was designed with two predefined endpoints. First, we assessed whether admission inflammatory biomarkers differed between patients with CT-positive traumatic findings and those with CT-negative mild traumatic brain injury. CT-positive intracranial trauma was defined as the presence of any acute traumatic intracranial abnormality on admission head computed tomography, including subdural hematoma, epidural hematoma, subarachnoid hemorrhage, intraparenchymal hemorrhage (cerebral contusion), or traumatic skull fracture. Patients without evidence of acute traumatic intracranial findings on CT were classified as CT-negative. Second, we evaluated differences in inflammatory biomarker levels across specific intracranial injury subtypes, with separate analyses performed for each lesion type. Patients with skull fractures and patients with negative CT scan were excluded from this analysis to isolate the effect of each intracranial lesion type on the examined biomarkers. Two separate sub-analyses were performed. The first included all patients with traumatic intracranial lesions. The second was restricted to patients with a single intracranial injury subtype, excluding those with multiple concurrent intracranial injuries, in order to evaluate the association between each specific trauma type and inflammatory biomarker levels.

### 2.6. Statistical Analysis

Univariate comparisons were performed using the independent-samples *t*-test or the Mann–Whitney U test for continuous variables and the chi-square test for categorical variables. Fisher’s exact test with Yates’ continuity correction was applied when expected cell counts were fewer than five or approached zero. To account for multiple testing within each biomarker, false discovery rate (FDR) correction was applied using the Benjamini–Hochberg method across the four hematoma subtype comparisons. Adjusted *p*-values (q-values) are reported. The association between time from injury to blood sampling and admission biomarker levels was assessed using Spearman’s rank correlation coefficient. Multivariable logistic regression analyses were performed including variables significant in univariable analyses and clinically relevant covariates. Statistical significance was defined as a *p*-value < 0.05.

## 3. Results

### 3.1. Baseline Characteristics

**Figure 1:**
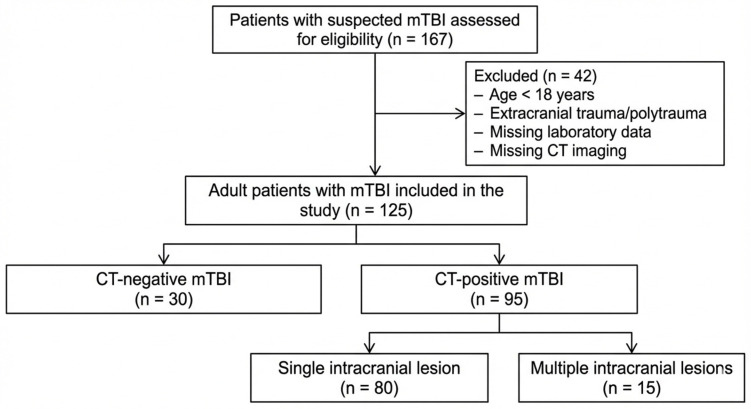
Flow Diagram of Patient Inclusion.

A total of 125 adult patients with mild traumatic brain injury were included in the analysis (Figure 1), of whom 95 (76%) were CT-positive and 30 (24%) were CT-negative. Among CT-positive patients, the most frequently observed intracranial injury patterns were cerebral contusions (*n* = 37) and subdural hematomas (*n* = 34), followed by subarachnoid hemorrhage (*n* = 29), and skull fractures (*n* = 15). Epidural hematoma was less common (*n* = 8). From these patients, multiple intracranial injuries were present in 15 patients, whereas most cases involved isolated lesions (*n* = 80). Baseline demographic and clinical characteristics are summarized in Table 1. Mean age did not differ between CT-positive and CT-negative patients (60.0 ± 21.5 vs. 57.1 ± 28.3 years, *p* = 0.953). The proportion of male patients was higher in the CT-positive group, although this difference did not reach statistical significance (56.83% vs. 43.3%, *p* = 0.214). The estimated time from injury to blood sampling was comparable between groups (84.1 ± 104.7 vs. 97.9 ± 173.8 min, *p* = 0.853). Spearman rank correlation analysis demonstrated no significant association between time from injury to blood sampling and PLR (ρ = −0.048, *p* = 0.90), GPR (ρ = −0.021, *p* = 0.95), or SII (ρ = 0.041, *p* = 0.91). There were no significant differences between CT-positive and CT-negative patients with respect to anticoagulant use, antiplatelet therapy, or recent alcohol or substance use. Falls were the most common mechanism of injury in the overall cohort (77 patients), followed by motor vehicle accidents (24 patients), falls from height (17 patients), and assault (7 patients). The distribution of injury mechanisms did not differ significantly between CT-positive and CT-negative patients (all *p* > 0.1), indicating a comparable mechanism profile between groups.

### 3.2. CT Positive vs. CT-Negative mTBI

Laboratory biomarkers stratified by CT status are presented as mean values with 95% confidence intervals in Table 2. No statistically significant differences were observed between CT-positive and CT-negative patients for PLR), GPR, and SII, all *p* > 0.05. In specific, admission PLR was comparable between CT-positive patients (159.19, 95% CI 137.82–180.55) and CT-negative patients (155.18, 95% CI 111.44–198.91; *p* = 0.793). Similarly, the GPR did not differ significantly between CT-positive (31.87, 95% CI 29.32–34.41) and CT-negative patients (29.50, 95% CI 23.18–35.81; *p* = 0.531). The SII also demonstrated higher values in CT-positive patients (1635.2, 95% CI 1272.1–1998.4) compared with CT-negative patients (1137.6, 95% CI 659.4–1615.8); however, this difference did not reach statistical significance (*p* = 0.291).

### 3.3. Comparison of Admission Inflammatory Indexes Across Intracranial Trauma Subgroups, Including Both Single and Multiple Lesions

Comparisons of admission inflammatory indexes in patients with single and multiple intracranial lesion types are summarized in Table 3. In the overall cohort, subdural hematoma was the only lesion type consistently associated with differences in routine laboratory parameters. Patients with subdural hematoma (SDH) demonstrated significantly higher GPR compared with those without SDH (mean 31.1 [95%CI 27.2–36.4] vs. 26.6 [22.6–32.7], *p* = 0.016). No statistically significant differences were observed for PLR or SII, nor for any biomarker with respect to intraparenchymal hemorrhage (contusion), subarachnoid hemorrhage (SAH), or epidural hematoma (EDH) (all *p* > 0.05). After application of Bonferroni correction for multiple comparisons, the associations between subdural hematoma and glucose-to-potassium ratio did not remain statistically significant. Similarly, no comparisons remained statistically significant following false discovery rate correction

A multivariable analysis was performed (4.9 Events-per-Variable) to evaluate whether SDH is independently associated with GPR levels. Multivariable logistic regression was conducted including the clinical and demographic variables presented in Table 1 and GPR levels. In this analysis, SDH remained a borderline significant independent predictor of higher GPR values (OR = 1.043, 95% CI: 1.022–1.064; *p* = 0.05). None of the remaining covariates showed a statistically significant association with GPR, including age (*p* = 0.25), sex (*p* = 0.55), anticoagulant use (*p* = 0.27), antiplatelet use (*p* = 0.26), time from injury to blood sampling (*p* = 0.53), or recent alcohol use or intoxication (*p* = 0.43).

### 3.4. Comparison of Admission Inflammatory Indexes Across Patients with Single Traumatic Intracranial Lesions

Analyses restricted to patients with single traumatic lesions (Table 3) yielded similar findings. Patients with isolated SDH exhibited higher PLR (213 [110–323] vs. 118 [87.6–179], *p* = 0.018) and GPR (33.3 [29.5–35.7] vs. 26.5 [22.4–31.7], *p* = 0.015), compared with patients without SDH. No significant associations were identified between any biomarker and contusion, SAH, or EDH in the isolated injury subgroup (all *p* > 0.05). Following Bonferroni adjustment, none of the observed associations in the isolated injury analysis remained statistically significant. Similarly, no comparisons remained statistically significant following false discovery rate correction.

A multivariable logistic regression analysis was performed (2.7 Events-per-Variable) in the subgroup of patients with single intracranial injuries to evaluate whether SDH was independently associated with GLR and PLR. The model included age, sex, time since injury, anticoagulant use, antiplatelet use, GPR, PLR, and recent alcohol use or intoxication. In this analysis, PLR remained independently associated with SDH (OR = 1.007, 95% CI 1.001–1.013; *p* = 0.025). None of the remaining covariates demonstrated a statistically significant association, including age (*p* = 0.85), sex (*p* = 0.76), anticoagulant use (*p* = 0.41), antiplatelet use (*p* = 0.08), GPR (*p* = 0.28), time since injury (*p* = 0.48), recent alcohol use or intoxication (*p* = 0.34).

### 3.5. Comparison of Admission Inflammatory Between Patients with Single vs. Multiple Traumatic Intracranial Lesions

To evaluate the influence of overall trauma burden on laboratory parameters, biomarker levels were compared between patients with mTBI and single lesions and those with multiple intracranial injuries. Patients with non-isolated trauma exhibited significantly higher inflammatory biomarker levels, including PLR, and SII compared with the group of single TBI lesions (Table 4). In contrast, GPR was higher in the non-isolated group but did not reach statistical significance (*p* = 0.087).

In multivariable analysis, including age, sex, time since injury, anticoagulant use, antiplatelet use, SII, PLR, and recent alcohol use or intoxication logistic regression analysis (2.5 Events-per-Variable), neither PLR nor SII remained independently associated (PLR: *p* = 0.403; SII: *p* = 0.505), despite both being significant in univariable analyses. Due to multicollinearity, PLR and SII were entered into separate models. None of the remaining covariates showed a statistically significant association.

## 4. Discussion

In this prospective study of patients with mTBI, routine admission inflammatory and metabolic indexes did not significantly differ between CT-positive and CT-negative patients, indicating limited utility for global intracranial injury discrimination. PLR, SII, and GPR were comparable between groups. When analyses were stratified by intracranial lesion subtype, SDH was the only lesion demonstrating a lesion-specific association with admission biomarkers. Specifically, SDH was associated with higher GPR in analyses including all intracranial injuries, while in the subgroup restricted to isolated lesions, SDH was additionally associated with higher PLR and GPR. However, after applying Bonferroni correction and false discovery rate adjustment, all these correlations became non-significant, indicating the preliminary and exploratory nature of the manuscript. No significant associations were observed between any biomarker and contusion, SAH, or EDH. Patients with multiple intracranial injuries exhibited higher PLR and SII compared with those with isolated lesions; however, in multivariable analyses, none of these associations remained significant.

Although none of the studied inflammatory indexes are Central Nervous System (CNS)-specific molecules, they appear to reflect the systemic stress response and activation of the sympathetic nervous system following injury [18,19]. Stress-induced hyperglycemia and neutrophil activation constitute only a limited component of the broader and highly complex host response to traumatic brain injury. Experimental studies have demonstrated that TBI triggers a robust neuroinflammatory cascade characterized by increased release of chemokines such as CXCL8/IL-8 and CCL21, as well as pro-inflammatory cytokines including IL-1, IL-6, IL-33, and TNF-α. Post-traumatic neutrophilia has been closely linked to elevated circulating levels of IL-8, which serves as a key chemotactic signal driving neutrophil activation and migration toward the site of injury [19,20,21,22]. Furthermore, neutrophil activation following brain trauma has been associated with astrocyte-derived cytokine release, including monocyte chemoattractant protein-1 (MCP-1/CCL2), which promotes the recruitment of macrophages and microglial cells to the injured parenchyma. These cells subsequently amplify the inflammatory response through the secretion of additional chemotactic mediators, thereby reinforcing neutrophil recruitment and activation [21,22,23]. The systemic increase in pro-inflammatory mediators, together with sustained microglial activation, contributes to disruption of the blood–brain barrier (BBB). This impairment facilitates the leakage of molecules released from damaged neurons and glial cells into the peripheral circulation, including GFAP, S100, and UCH-L1, which currently represent the principal blood-based biomarkers of TBI [24,25,26,27,28].

Compared with the measurement of interleukins, assessing the NLR, PLR, and other peripheral blood cell populations is simpler, faster, and can be performed using a standard hematology analyzer, which is available in almost all healthcare facilities [14]. In addition, a potential association appears to exist between IL levels and neutrophil-related indexes in patients with TBI, as demonstrated in an analysis of patients with concussion, where NLR and IL-6 levels showed a near-statistically significant correlation (r = 0.31, 95% CI −0.03 to 0.58, *p* = 0.06) [26]. The NLR has been investigated in recent years as a biomarker for identifying intracranial injury in mild TBI, as well as for prognostication in patients with more severe TBI. In a study including 478 patients with TBI, NLR was found to be significantly associated with the modified Rankin Scale (mRS) score at 6 months after injury, as well as with mortality [28]. In another pediatric study involving 82 patients with TBI, NLR was associated with injury severity and overall prognosis [27]. With respect to the association between NLR and the need for neurosurgical intervention, a study of 1077 patients with cerebral contusions demonstrated that NLR was significantly associated with early contusion expansion and the requirement for surgical management [29]. Beyond NLR, other inflammatory indexes have also demonstrated prognostic value in patients with TBI, including the PLR and the SII. PLR has been associated with outcomes in patients with moderate and severe TBI [30,31,32]. The prognostic relevance of platelet-based inflammatory indices in TBI has been increasingly explored, particularly in populations with more severe injury. A recent systematic review by Ilyas et al. [30] demonstrated that elevated platelet-to-lymphocyte ratio (PLR) is consistently associated with poorer clinical outcomes in adult TBI, although substantial heterogeneity was noted across studies in terms of injury severity, timing of sampling, and outcome measures. Similarly, Li and Deng [31] reported that higher admission PLR independently predicted short-term mortality in patients with moderate-to-severe TBI, underscoring the potential value of PLR as a marker of systemic inflammatory burden in more severe injury states. In addition, Arslan and Sahin [32] evaluated multiple inflammatory indices and found that SII, NLR, and PLR were all associated with adverse outcomes following TBI. In contrast to these findings, our study, focused exclusively on mTBI, did not demonstrate a significant association between PLR or SII and overall CT positivity, suggesting that the prognostic and discriminatory utility of platelet-based indices may be attenuated in lower-severity TBI. Furthermore, in the study by Wang et al., PLR, in combination with NLR, was associated with the progression of cerebral contusion size and hemorrhage expansion [33]. Finally, in a study conducted at the same institution where the present work was performed, NLR was identified as a potential biomarker that may contribute to the early recognition of this condition, with a sensitivity of approximately 90% [34].

The SII is an index with a biological background similar to that of NLR and PLR, as it reflects the organism’s stress response, while the magnitude of its alteration appears to correlate with disease severity and prognosis under various inflammatory conditions [35]. With regard to TBI, SII has only recently begun to be investigated as a potential biomarker. In a recent study including 102 patients, SII was directly associated with TBI prognosis, and its diagnostic performance was superior to that of NLR (AUC: 0.845 vs. 0.694) [25]. Similar findings have been reported in other studies, which also demonstrated an association between SII and injury severity [32,36,37,38]. However, in a pediatric study of 206 patients, although SII was associated with TBI severity, it was not found to be related to final outcome (death vs. survival) [36].

Elevated blood glucose levels are a common finding in patients with TBI, both on arrival at the emergency department and during hospitalization. TBI-related hyperglycemia, similar to neutrophilia, reflects the systemic stress response. Physiological stress leads to activation of the sympathetic nervous system and stimulation of the hypothalamic–pituitary–adrenal axis, resulting in the release of catecholamines, cortisol, and growth hormone, as well as enhanced hepatic gluconeogenesis, which collectively increase blood glucose levels [39]. An additional mechanism associated with hyperglycemia in patients with TBI is increased insulin resistance, secondary to the release of cytokines such as IL-1 and TNF-α [39,40]. Under physiological conditions, this stress-induced hyperglycemia aims to meet the increased energy demands of peripheral organs and to preferentially channel glucose to priority organs (brain, heart) through modulation of insulin resistance. However, excessively high glucose levels have been associated with adverse outcomes in patients with TBI. Specifically, admission hyperglycemia in patients with TBI has been linked to increased mortality and morbidity, as well as poorer functional and neurological outcomes [41,42,43,44,45,46]. In the present study, the GPR was associated with the presence of subdural hematoma (SDH); however, it did not discriminate between CT-positive and CT-negative patients overall. In contrast, Wang et al. [14] reported GPR to be an independent prognostic factor in patients with mild-to-moderate traumatic brain injury. This discrepancy may be partly attributable to differences in study populations, particularly the inclusion of patients with moderate TBI in the cohort studied by Wang et al., which may have amplified the prognostic relevance of GPR [47].

The observation that SDH could be correlated with higher admission inflammatory/metabolic indexes is biologically plausible and, if validated in larger cohorts, may be hypothesis-generating for a meningeal-driven component of the early systemic response. Because SDH develops in close anatomic proximity to the arachnoid and leptomeninges, blood products and local tissue stress could preferentially activate meningeal immune pathways and secondarily into peripheral counts-based indexes, even when parenchymal injury is limited [48]. This raises the possibility that SDH is a lesion subtype in which leptomeningeal biomarkers such as meningeal-enriched cytokines/chemokines, endothelial–leukocyte trafficking markers, or other meningeal inflammatory signatures in blood and CSF could add mechanistic specificity beyond NLR/PLR/SII. Importantly, this biomarker space remains relatively underexplored in mTBI/SDH and warrants targeted, lesion-stratified studies with paired systemic and CSF sampling and longitudinal profiling.

Previous studies on blood-based biomarkers have demonstrated potential associations between traumatic intracranial lesions and the need for urgent neurosurgical intervention, as well as with the number and extent of lesions in patients with TBI [49,50,51]. Given that the majority of urgent surgical lesions causing deterioration in the level of consciousness in patients with mTBI are rapidly enlarging extra-axial hematomas, these findings suggest the presence of a distinct biological response for different types of intracranial trauma. In a study including 215 patients across all severities of TBI, Okonkwo et al. observed a significant correlation between the number of traumatic intracranial lesions on CT and GFAP levels [49]. Similarly, in our study, patients with multiple intracranial injuries exhibited higher PLR and SII values compared with patients with a single intracranial injury. In another study of 324 patients with TBI, Jussi et al. demonstrated that GFAP and UCH-L1 could discriminate between patients with mass lesions and those with diffuse injuries, further supporting a distinct response profile for each type of intracranial injury [50]. However, lesion-specific biomarker analyses remain limited, and further studies are warranted to determine whether distinct inflammatory profiles can reliably characterize individual intracranial lesion subtypes.

This study has several limitations. Although data were prospectively collected, the overall sample size was modest, particularly within individual intracranial lesion subgroups, limiting statistical power and likely contributing to the loss of statistical significance after Bonferroni correction despite consistent effect directionality, especially for subdural hematoma-associated findings. Across lesion-specific multivariable logistic regression models, events per variable (EPV) ranged from approximately 2.5 to 5.0. Given these EPV values, all adjusted analyses should be interpreted as exploratory and hypothesis-generating. Inflammatory biomarkers were measured at a single admission time point, precluding assessment of temporal inflammatory dynamics after injury. Despite exclusion of patients with major extracranial trauma, diabetes mellitus, and hematologic disorders, residual confounding from unmeasured physiological stressors, comorbidities, or medications cannot be excluded. The exclusive use of routine systemic biomarkers limits mechanistic inference regarding central or meningeal inflammation, and the single-center design may restrict generalizability, underscoring the need for multicenter validation and longitudinal biomarker assessment.

## 5. Conclusions

In this prospective cohort of patients with mTBI, routinely available admission inflammatory and metabolic indices showed limited utility for differentiating CT-positive from CT-negative cases. When analyses were performed at the level of individual intracranial lesion types, SDH was the only lesion demonstrating a potential, albeit moderate, association with higher admission PLR and GPR, whereas no significant associations were observed for other traumatic intracranial injury types. However, after statistical adjustment for multiple comparisons, none of these parameters remained significant, indicating the exploratory nature of the study and requiring further investigation in larger cohorts to clarify whether SDH is associated with distinct systemic inflammatory responses.

## Figures and Tables

**Table 1:** Baseline characteristics of the study population.

Variable	All Patients (*n* = 125)	CT-Positive (*n* = 95)	CT-Negative (*n* = 30)	*p* Value
Age, years, mean ± SD	59.6 ± 22.6	60.0 ± 21.5	57.1 ± 28.3	0.953
Male sex, n (%)	67 (53.6%)	54 (56.8%)	13 (43.3%)	0.214
Estimated time since injury (min), mean ± SD	85.9 ± 110.2	84.1 ± 104.7	97.9 ± 173.8	0.853
Anticoagulant use, n (%)	21 (16.8%)	18 (18.9%)	3 (10.0%)	0.401
Antiplatelet use, n (%)	19 (15.2%)	17 (17.9%)	2 (6.7%)	0.241
Recent alcohol/substance use, n (%)	24 (19.2%)	21 (22.1%)	3 (10.0%)	0.187

**Table 2:** Laboratory inflammatory and metabolic biomarkers according to CT findings.

Biomarker	CT-Positive (95% CI)	CT-Negative (95% CI)	*p* Value
PLR	159.19 (137.82–180.55)	155.18 (111.44–198.91)	0.793
GPR	31.87 (29.32–34.41)	29.50 (23.18–35.81)	0.531
SII	1635.2 (1272.1–1998.4)	1137.6 (659.4–1615.8)	0.291

PLR, platelet-to-lymphocyte ratio; SII, systemic immune-inflammation index (platelet count × neutrophil count/lymphocyte count); GPR, glucose-to-potassium ratio.

**Table 3:** Admission biomarkers according to trauma subtype.

Lesion Subtype	Cohort	Biomarker	Lesion Present (Mean, 95% CI)	Lesion Absent (Mean, 95% CI)	*p* Value	q Value (FDR)
Contusion	All lesions:	PLR	125 (84.7–165)	131 (91.1–205)	0.419	0.838
		GPR	28.9 (24.4–38.6)	28.0 (23.8–33.4)	0.392	0.784
		SII	1503 (551–2100)	957 (621–2040)	0.968	0.968
	Single lesions only:	PLR	164 (95.5–306)	118 (87.6–179)	0.842	0.903
		GPR	30.7 (26.2–37.1)	26.8 (22.4–33.3)	0.786	0.987
		SII	1740 (606–3949)	1024 (598–1974)	0.500	0.858
Subdural hematoma (SDH)	All lesions	PLR	213 (125–303)	118 (87.6–179)	0.061	0.244
		GPR	31.1 (27.2–36.4)	26.6 (22.6–32.7)	0.016	0.064
		SII	1760 (957–2832)	1024 (598–1974)	0.071	0.284
	Single lesions only:	PLR	213 (110–323)	118 (87.6–179)	0.018	0.072
		GPR	33.3 (29.5–35.7)	26.5 (22.4–31.7)	0.015	0.060
		SII	1975 (1348–3823)	1024 (598–1974)	0.064	0.256
Subarachnoid hemorrhage (SAH)	All lesions:	PLR	120 (90.4–198)	131 (87.6–205)	0.711	0.903
		GPR	29.3 (24.4–41.0)	28.0 (23.3–33.3)	0.528	0.704
		SII	1024 (598–2810)	957 (621–2040)	0.571	0.968
	Single lesions only:	PLR	118 (87.6–198)	131 (87.6–205)	0.590	0.903
		GPR	30.7 (24.7–41.3)	28.1 (23.3–33.3)	0.512	0.987
		SII	830 (598–2100)	957 (621–2040)	0.721	0.858
Epidural hematoma (EDH)	All lesions:	PLR	131 (96.7–282)	125 (90.1–184)	0.903	0.948
		GPR	25.6 (22.4–30.9)	28.3 (23.8–35.6)	0.987	0.987
		SII	1191 (599–2910)	1024 (598–2100)	0.858	0.968
	Single lesions only:	PLR	131 (96.7–282)	125 (90.1–184)	0.903	0.903
		GPR	25.6 (22.4–30.9)	28.3 (23.8–35.6)	0.987	0.987
		SII	1191 (599–2910)	1024 (598–2100)	0.858	0.858

Abbreviations: PLR, platelet-to-lymphocyte ratio; SII, systemic immune-inflammation index (platelet count × neutrophil count/lymphocyte count); GPR, glucose-to-potassium ratio. All values represent comparisons between patients with the specified lesion subtype and all remaining patients in the sub-cohort without that lesion. q = False Discovery Rate-adjusted *p*-value.

**Table 4:** Comparison of admission inflammatory between patients with single vs. multiple traumatic intracranial lesions.

Biomarker	Single Intracranial Trauma (95%CI)	Multiple Intracranial Traumas (95%CI)	*p*-Value
PLR	118 (87.6–179)	213 (110–323)	0.012
SII	1024 (598–1974)	1760 (957–2832)	0.021
GPR	26.8 (22.4–33.3)	28.9 (24.4–38.6)	0.087

Abbreviations: PLR, platelet-to-lymphocyte ratio; SII, systemic immune-inflammation index (platelet count × neutrophil count/lymphocyte count); GPR, glucose-to-potassium ratio.

## Data Availability

The original contributions presented in this study are included in the article. Further inquiries can be directed to the corresponding author.

## Abbreviations

The following abbreviations are used in this manuscript:

BBB	Blood–brain barrier
CDR	Clinical decision rule
CI	Confidence interval
CT	Computed tomography
EDH	Epidural hematoma
GCS	Glasgow Coma Scale
GPR	Glucose-to-potassium ratio
IL	Interleukin
mTBI	Mild traumatic brain injury
NLR	Neutrophil-to-lymphocyte ratio
PECARN	Pediatric Emergency Care Applied Research Network
PLR	Platelet-to-lymphocyte ratio
SAH	Subarachnoid hemorrhage
SDH	Subdural hematoma
SII	Systemic immune-inflammation index
TBI	Traumatic brain injury
